# Histoacryl glue injection in walled-off necrosis impression on gastric wall mistaken as gastric varix: an unintended adverse event having therapeutic benefit

**DOI:** 10.1016/j.igie.2023.11.005

**Published:** 2023-12-01

**Authors:** Kishalaya, Dipankar Mondal, Mousam Dey, Souveek Mitra, Srijan Mazumdar

**Affiliations:** Department of Gastroenterology and Hepatology, Indian Institute of Liver & Digestive Sciences, Kolkata, India

A 46-year-old man with history of ethanol use disorder was admitted in a secondary health center with a 4-week history of abdominal pain followed by progressive development of an upper abdominal lump. CT imaging performed after admission showed a large walled-off necrotic collection containing blood in the lesser sac (Fig. 1). The patient experienced melena during the hospital stay. He underwent EGD, in which a large varix was reported in proximal gastric body (Fig. 2), and Histoacryl glue injections (4 mL and 2 mL on consecutive days; Tandem Life Sciences, Hyderabad, India) were injected. He continued to experience melena and was subsequently referred to a tertiary care center for better management.

**Figure 1:**
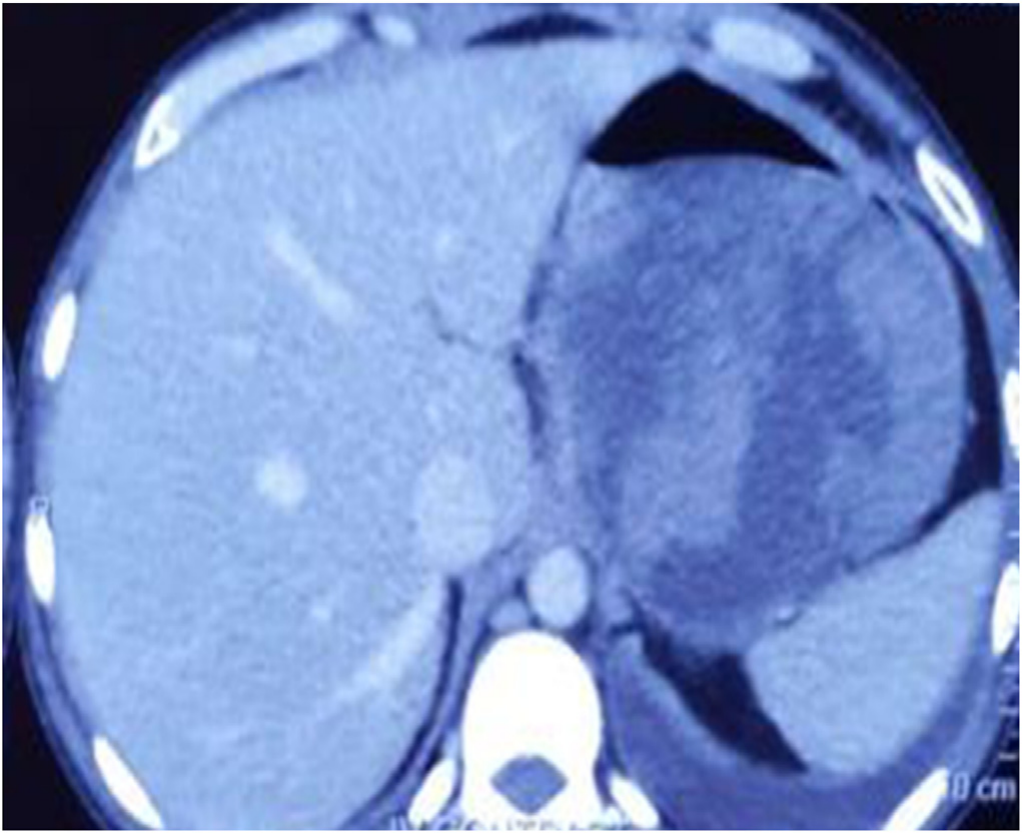
Walled-off necrotic collection in the lesser sac.

**Figure 2:**
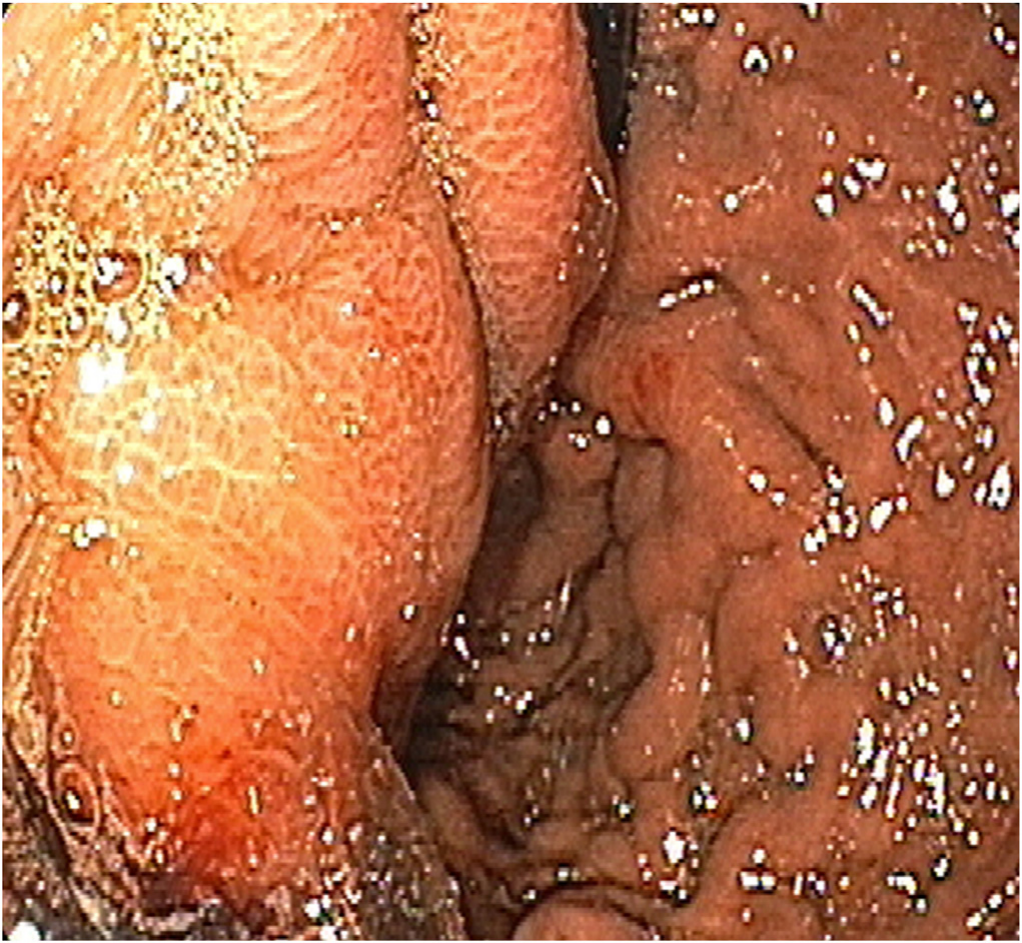
Walled-off necrotic impression on gastric wall mistaken as varix.

Upon admission at our center, the patient’s clinical examination revealed no lump in his upper abdomen. EGD showed an opening in the proximal gastric body communicating with a closed space (Fig. 3).

**Figure 3:**
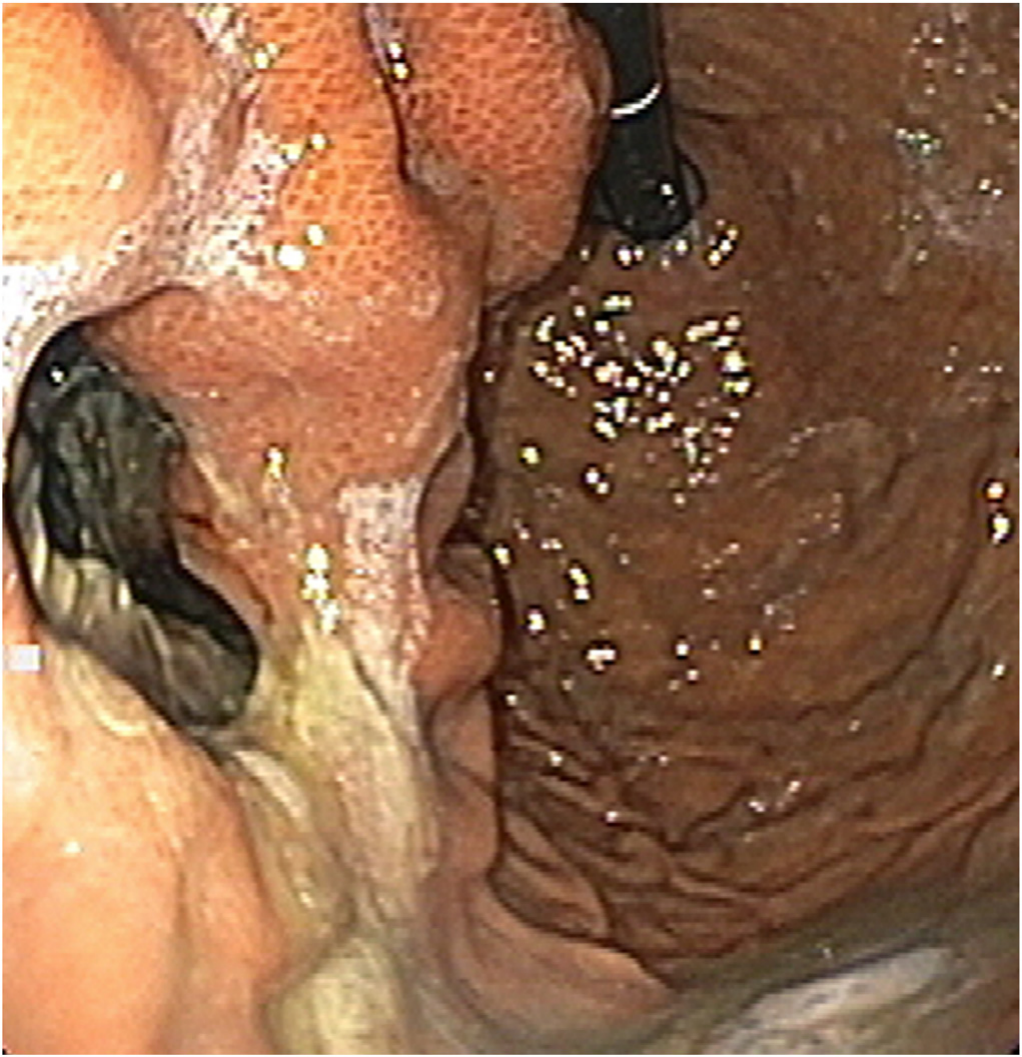
Wall defect in proximal stomach.

Upon retrospection, we concluded that the previous endoscopist had mistaken the walled-off necrosis (WON) impression for a gastric varix and performed the Histoacryl injections. The scope in retroflexed position was showing the bulge caused by the WON compression from outside and not a true gastric varix (Fig. 4). Necrosis induced by the walled off necrotic contents (mainly pancreatic enzymes), pressure tamponading by the WON, glue needle–induced trauma to the gastric wall, and necrosis induced by Histoacryl glue retained within the gastric wall led to rupture of the walled off necrotic contents into the gastric lumen and subsequent disappearance of the abdominal lump.

**Figure 4:**
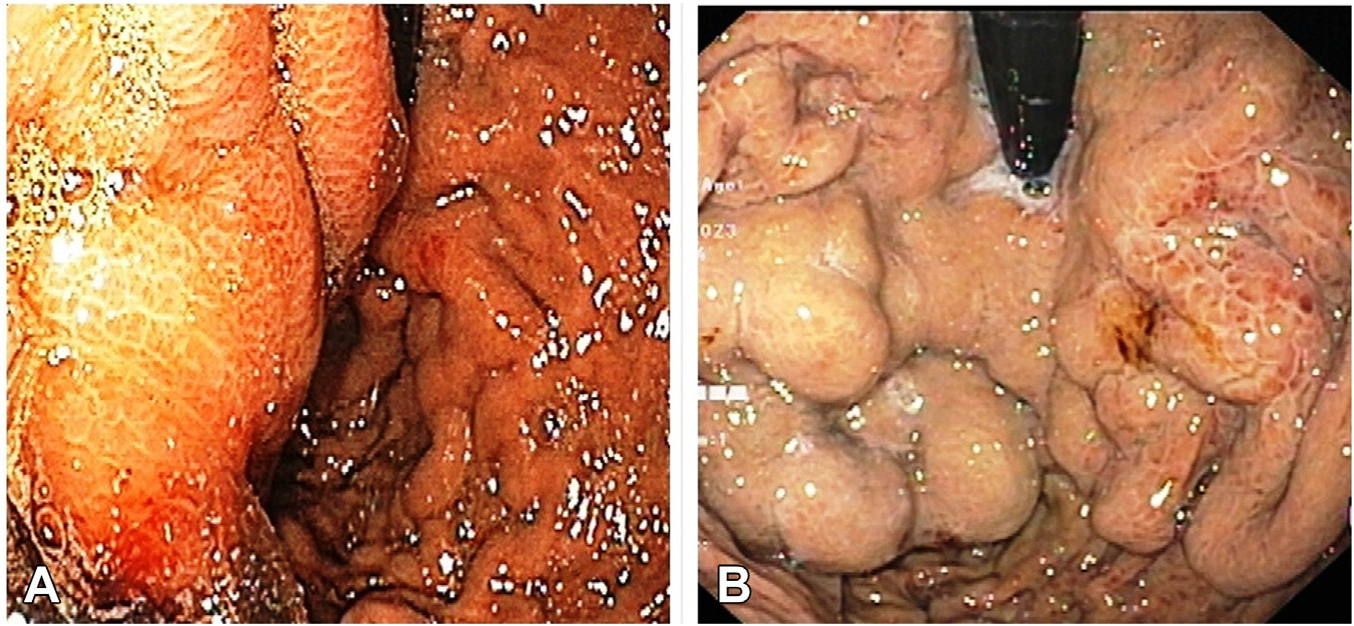
Walled-off necrotic impression mistaken as a varix in the current case (**A**) and true gastric fundal varices (**B**).

We repeated CT scan at our center. It showed almost complete resolution of WON (Fig. 5) along with a 2 mm pseudoaneurysm in a proximal branch of the splenic artery.

**Figure 5:**
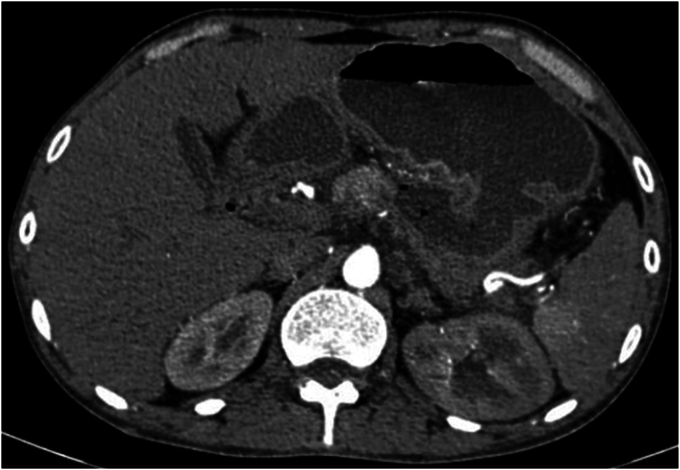
Resolving walled-off necrosis.

The patient again experienced hematemesis. Coil embolization of the pseudoaneurysm was performed, and the patient was discharged. CT imaging at 8 weeks’ follow-up showed no intra-abdominal collection, no flow in the aneurysm, and preserved splenic perfusion.

## Discussion

Spontaneous rupture of WON into the GI lumen is a rare occurrence, with only a few reported cases.^1^, ^2^, ^3^ Development of a pseudoaneurysm after an attack of acute pancreatitis occurs in <10% of cases.^4^ Splenic artery is the most common site of pseudoaneurysm formation.^5^ Pseudoaneurysms are known to be gifted masqueraders in terms of their clinical presentation. We report a case in which the patient developed a symptomatic WON and a pseudoaneurysm after an attack of acute pancreatitis. Both WON and the pseudoaneurysm were symptomatic in the form of pressure symptoms and upper GI bleeding, respectively. Spontaneous rupture of WON into the stomach was facilitated by glue injections in the gastric bulge (mistaken as varix) caused by WON compression from outside; identification of pseudoaneurysm as the cause of the upper GI bleeding and subsequent management were done later in the course of the patient’s illness.

CT imaging of the patient’s abdomen performed at our center showed a pseudoaneurysm in a proximal branch of the splenic artery, which was not reported in the CT scan done at the previous center. Upon retrospection, we concluded that both episodes of GI bleeding (melena initially followed by hematemesis) were caused by the pseudoaneurysm. The initial bout of melena caused by the pseudoaneurysm stopped spontaneously. The endoscopist at the previous center was not properly aware of the patient’s history, and they mistook the WON impression as gastric varix and performed Histoacryl glue injections. This misdiagnosis was due to a combination of not properly knowing the patient’s medical history and poor understanding of gastric varices. Subsequent rupture of WON into the gastric lumen and expulsion of its content rectally led to the impression of ongoing melena and failure to control bleeding. This prompted the referral of the patient to a higher center. The second bout of bleeding in the form of hematemesis was also caused by the pseudoaneurysm. This sequence of events was realized in retrospection, and coil embolization of the pseudoaneurysm was performed.

## Conclusion

In conclusion, along with adequate experience and expertise, a clinician must have proper knowledge of patient’s history before performing a therapeutic procedure. Hasty and improperly informed decisions often lead to adverse events. Fortunately, in our case, one such adverse event benefited the patient. Also, splenic artery pseudoaneurysms, well-known masqueraders in their clinical presentation, should always be kept as a differential in patients with acute pancreatitis presenting with upper GI bleeding.

## Disclosure

All authors disclosed no financial relationships.
